# Is a Gluten-Free Diet Enough to Maintain Correct Micronutrients Status in Young Patients with Celiac Disease?

**DOI:** 10.3390/nu12030844

**Published:** 2020-03-21

**Authors:** Teresa Nestares, Rafael Martín-Masot, Ana Labella, Virginia A. Aparicio, Marta Flor-Alemany, Magdalena López-Frías, José Maldonado

**Affiliations:** 1 Department of Physiology and “José MataixVerdú” Institute of Nutrition and Food Technology (INYTA), University of Granada, 18071 Granada, Spain; virginiaparicio@ugr.es (V.A.A.); martadelaflor@correo.ugr.es (M.F.-A.); maglopez@ugr.es (M.L.-F.); 2 Pediatric Gastroenterology and Nutrition Unit, Hospital Regional Universitario de Malaga, 29010 Málaga, Spain; rafammgr@gmail.com; 3 Pediatric Clinical Management Unit., “San Cecilio” University Hospital, 18016 Granada, Spain; alnestares92@gmail.com; 4 Sport and Health University Research Institute (iMUDS), 18071 Granada, Spain; 5 Department of Pediatrics, University of Granada, 18071 Granada, Spain; jmaldon@ugr.es; 6 Biosanitary Research Institute, 18071 Granada, Spain; 7 Maternal and Child Health Network, Carlos III Health Institute, 28029 Madrid, Spain; 8 Pediatric Clinical Management Unit. “Virgen de las Nieves” University Hospital, 18071 Granada, Spain

**Keywords:** celiac disease, gluten-free diet, nutritional adequacy, iron deficiency anemia, children

## Abstract

The current study assesses whether the use of a gluten-free diet (GFD) is sufficient for maintaining correct iron status in children with celiac disease (CD). The study included 101 children. The celiac group (*n* = 68) included children with CD, with long (> 6 months) (*n* = 47) or recent (< 6 months) (*n* = 21) adherence to a GFD. The control group (*n* = 43) included healthy children. Dietary assessment was performed by a food frequency questionnaire and a 3-day food record. Celiac children had lower iron intake than controls, especially at the beginning of GFD (*p* < 0.01). The group CD-GFD >6 months showed a higher intake of cobalamin, meat derivatives and fish compared to that of CD-GFD <6 months (all, *p* < 0.05). The control group showed a higher consumption of folate, iron, magnesium, selenium and meat derivatives than that of children CD-GFD >6 months (all, *p* < 0.05). Control children also showed a higher consumption of folate and iron compared to that of children CD-GFD <6 months (both, *p* < 0.05). The diet of celiac children was nutritionally less balanced than that of the control. Participation of dietitians is necessary in the management of CD to guide the GFD as well as assess the inclusion of iron supplementation and other micronutrients that may be deficient.

## 1. Introduction

Celiac disease (CD) occurs in about 1% of people in most populations [1,2]. Diagnosis rates are increasing, which seems to be due to a true rise in incidence that requires greater awareness and early detection. In response to unknown environmental factors, it is believed that ingestion of gluten promotes an immunologically mediated small intestinal enteropathy in genetically susceptible individuals [3]. The disease primarily affects the small intestine; however, the clinical manifestations are large, with both intestinal and extra-intestinal symptoms. Patients with CD might also present with various deficiency states, including anemia, osteopenia or osteoporosis. Upon diagnosis, patients should be tested, since between 20%–38% of CD patients show nutritional deficiencies. Specifically, 12%–69% display iron deficiency, more frequent in adults than in children [4], and 8%–41% display Vitamin B12 deficiency, and about 31% of patients with CD present low ferritin concentrations [5].

A lifelong strict gluten-free diet (GFD) is the only available treatment for CD. Improvement and resolution of symptoms typically occur within days or weeks, and often precede the normalization of serological markers and of duodenal villous atrophy [6]. About 20% of patients with CD have persistent or recurrent symptoms despite having a good adherence to a GFD [7], and the nutritional adequacy of the GFD remains controversial [8], although evidence suggests that GFD is nutritionally unbalanced [8]. In fact, wheat is a source of iron, folates and B vitamins (thiamin, riboflavin and niacin) and not only a major source of protein. Indeed, gluten-free products are low in these nutrients compared to their gluten-containing equivalents [9,10,11].

An essential micronutrient for adequate erythropoietic function is iron, which is implicated in oxidative metabolism, enzymatic activities and cellular immune responses [12]. Iron deficiency anemia (IDA) is a public health problem where children are one of the weaker groups [13]. CD leads to decreased absorption of many nutrients in duodenum-mucosal damage, including iron [14] (the site of maximal iron absorption). Therefore, iron malabsorption is usually observed in CD and may be the presenting clinical feature even in the absence of weight loss or diarrhea [15,16,17]. Although IDA is very common in CD, it could persist after the initiation of a GFD [18]. Unfortunately, the relationship between IDA and CD has been poorly explored.

Therefore, the purpose of the present study was to determine the importance of a GFD in the normalization of iron metabolism in patients with CD, as well as to test the influence of the time in a GFD on iron metabolism.

## 2. Materials and Methods

### 2.1. Subjects

The study was carried out according to the principles of the Declaration of Helsinki and its later amendments and approved by the Ethics Committee of the University of Granada (Ref. 201202400000697). The study included 101 children aged 7–18 years old, attending the Gastroenterology, Hepatology and Child Nutrition Service from the “Virgen de las Nieves” University Hospital in Granada, Spain. 

The control group included 43 healthy children, whose serological screening was detected to be negative and who had no history of any chronic disease. These children attended this Service due to minor symptoms related to chronic functional constipation, according to the Rome IV criteria. After verifying that it was due to transitory gastrointestinal symptoms (functional constipation), they were included in the control group. The inclusion criteria for the control group were age between 7 and 18 years, absence of serum IgA and IgG anti-transglutaminase (tTG) antibodies, normal weight for the age, absence of gastrointestinal disorders in the previous year and normal appetite. Children with CD diagnosed in accordance with the European Society for Pediatric Gastroenterology Hepatology and Nutrition (ESPGHAN) [19] were included in the CD group (*n* = 68), which was divided into patients on a GFD longer than 6 months (GFD > 6, *n* = 47) and patients following a GFD less than 6 months (GFD < 6, *n* = 21). The exclusion criteria for both groups were liver or kidney diseases, acute and chronic inflammation, inflammatory bowel disease, diabetes, chronic asthma and consumption of dietary supplements containing substances with antioxidant activity. We also excluded obese patients (according to the criteria of the International Task Force) [20] and those who did not sign the informed consent. Written informed consent was obtained from all parents.

### 2.2. Clinical and Socio-Demographics

Participants’ clinical and socio-demographic characteristics including age, household composition, parents’ marital status, educational level and smoking habit were assessed by the same group of researchers.

When IDA was detected in CD children, elemental iron was administered at doses of 3 to 5 mg per kilogram of weight per day. When iron deficiency without anemia was detected, prophylactic doses of elemental iron were prescribed at a dose of 2 mg per kilogram per day in two daily doses for 2–3 months [21].

### 2.3. Anthropometric Measures

Anthropometric characteristics (weight, height) were assessed in the control and the celiac subjects. Height was measured to the nearest 5 mm using a stadiometer (Seca 22, Hamburg, Germany). Body weight was measured using the same mechanical balance (Seca 200, Hamburg, Germany).

### 2.4. Blood Haematological and Biochemical Anaemia-Related Markers

Venous blood samples were collected into anticoagulated tubes with sodium heparin during morning hours from fasting patients. In order to obtain plasma, blood samples were centrifuged at 2500× *g* at 4 °C for 10 min. Plasma samples were frozen (−80 °C) until measurements. All hematological and biochemical parameters studied were measured using an automated hematology analyzer (K-1000D, Sysmex, Tokyo, Japan). The following biochemical parameters were determined: iron, ferritin, transferrin, thyroid-stimulating hormone (TSH) and thyroxine.

### 2.5. Dietary Assessment

Dietary intake was assessed through a semi-quantitative 78-item food frequency questionnaire (FFQ) previously validated in Spain by Mataix et al. [22] and a three-day food record, two on weekdays and one on the weekend. The diary was carefully explained, face to face, by the same trained dietitian to the children and their parents and was accompanied by detailed instructions for the compilation and a photographic atlas including different portion size pictures and a set of household measures [23]. The survey included a daily record of all foods consumed during the different meals (breakfast, morning snack, lunch, afternoon snack, dinner). For each meal, participants were requested to report an exhaustive description of the food and the recipes (including cooking methods and sugar or fats added during the meal preparation), food amount (according to the atlas) and the brands of packaged foods consumed.

All diaries were analyzed by the same trained dietitian using the Evalfinut software that includes the Spanish Food Composition Database [24]. The program estimated the energy intake (kilocalories) and macronutrients (proteins, total fats, saturated fats, carbohydrates, simple sugars and fiber, expressed in grams) and the percentage of energy provided by each macronutrient. Recommended Energy and Nutrient Intake Levels for Spanish population [24] were taken as reference values for energy and nutrient intake and for food group consumption. The composition of GF products was retrieved from product labels.

### 2.6. Data Analyses 

We employed descriptive statistics (mean, standard deviation) for quantitative variables and percentage of participants (%) for categorical variables to describe the baseline characteristics of the study sample. We conducted Student’s *t*-test to explore differences in the continuous variables. Furthermore, we assessed differences in categorical variables by using the chi-squared test. 

A one-way analysis of covariance (ANCOVA) after adjustment for age, sex and body weight was employed to assess the differences in hematological and biochemical anemia-related markers between the celiac and control healthy groups. An ANCOVA after adjustment for age, sex and body weight was also performed to explore differences in hematological and biochemical anemia-related markers and dietary behavior between the healthy controls, the patients with a GFD in the last 6 months and the patients engaged in a GFD less than 6 months. Significant pairwise comparisons with Bonferroni adjustment were performed to keep the experiment error rate at α = 0.05 and to identify between which groups the differences were significant (e.g., control healthy group vs. less than 6 months in a GFD). 

The data analyses were conducted with the Statistical Package for Social Sciences (IBM SPSS Statistics for Windows, Version 20, IBM Corp, Armonk, NY, USA), and statistical significance was set at *p ≤* 0.05.

## 3. Results

The baseline sociodemographic and anthropometric characteristics of the study sample are shown in Table 1. A total of 68 children with CD participated in the study (mean age 8.5 ± 4.1 years). More than half of the participants with CD followed a GFD for more than 6 months and met physical activity recommendations. The control group included 43 healthy children (mean age 10.3 ± 4.5 years). There were differences in height (*p* = 0.006) and weight (*p* = 0.002) between groups. Regarding the hematological results, the CD group showed lower levels of hemoglobin, erythrocyte and hematocrit compared to healthy children (*p* < 0.05).

Differences in serum hematological and biochemical anemia-related markers between healthy control children, patients with a GFD in the last 6 months and patients with a GFD less than 6 months are shown in Table 2. Serum erythrocytes differed between groups (*p* = 0.016), with pairwise comparisons showing lower erythrocyte counts in both groups of CD patients compared to the healthy control group (*p* < 0.05). Hematocrit also differed between groups (*p* = 0.031), with pairwise comparisons showing lower hematocrit concentration in the CD group following the GFD for less than 6 months compared to the control group (38.6 ± 0.7 versus 41.0 ± 0.4, respectively, *p* < 0.05). Finally, hemoglobin levels differed between groups (*p* < 0.05), being lower in CD groups but without pairwise significant difference.

Table 3 and Table 4 show the total daily energy and the macronutrient and micronutrient intakes in the three study groups, and the results from daily food group consumption, as collected by the FFQ. Total energy daily intake did not differ between groups. There were no significant differences for macronutrient intake. There were some differences for micronutrients involved in iron metabolism; iron intake was lower for CD groups vs controls (*p* < 0.006). Selenium and magnesium intake was lower for GFD > 6 months vs the control group (*p* < 0.001). 

The differences in dietary habits of the study participants between the control group, the celiac children following a GFD for at least 6 months and the celiac children following a GFD for less than 6 months are shown in Table 3. The children with CD who followed a GFD for at least 6 months showed a higher intake of cobalamin, meat derivatives and fish when compared with those patients who followed a GFD for less than 6 months (all, *p* < 0.05). The children in the healthy control group showed a higher consumption of folate, iron, magnesium, selenium and meat derivatives than those children with CD following a GFD for at least 6 months (all, *p* < 0.05). The children in the control group showed a higher consumption of folate and iron compared with the children with CD following a GFD for less than 6 months (both, *p* < 0.05).

## 4. Discussion

The main findings of the present study indicate that patients with CD showed lower levels of hemoglobin, erythrocyte and hematocrit compared to healthy children, although neither group presented average values compatible with anemia, nor microcytosis. Regardless of the GFD tracking time, those patients following a GFD for more than 6 months showed a lower hematocrit than healthy controls. Moreover, both CD groups had lower iron and folate intake than the healthy controls. In addition, a lower intake of magnesium and selenium was also observed in patients with CD who had followed a GFD for more than 6 months compared to the control group. Finally, the children with CD who had followed a GFD for less than 6 months showed a lower cobalamin intake compared to those with a greater period of adherence.

In our study, there were 52 (76.5%) celiac females vs 20 (46.5%) healthy females, a sample that is representative of the real incidence of CD by sex [25] and consistent with other similar ones [26]. Moreover, the groups differed in age and weight, but we controlled all the analyses for such covariates.

It is well known that CD is a cause of anemia as a result of malabsorption of iron, folic acid and cobalamin [5]. IDA is a frequent finding in patients with CD and adherence to a GFD may be sufficient to reverse IDA and iron deficiency, although both may persist despite a good adherence to a GFD [7,23]. In fact, in the present study, erythrocyte count, hemoglobin and hematocrit were lower in CD patients compared to healthy controls. However, these patients cannot be considered anemic (despite hemoglobin was lower in CD groups, these values were within the normal range for its age). It is possible that the non-presence of IDA in our patients with CD may be due to the fact that, in addition to the GFD, all children who presented IDA at the time of the diagnosis of CD were prescribed oral iron supplements (ferrous sulfate). This fact could induce a normalization of both the IDA and ID indicators in the first months of treatment, despite the fact that dietary iron intake was lower than recommended [22]. A strict monitoring of the GFD leads to the remission of clinical symptoms, the normalization of serological markers of CD and the recovery of the histological lesion in the small intestine, but the IDA and/or the ID may take a long time in recovering, between 6 months and a year or even longer [17,23]. In addition, it should be taken into account that anemia is present in 22%–63% of patients with CD at the time of diagnosis [15]; the more severe the villus lesion of the intestine, the higher the incidence of anemia [27].

In addition to ID, it is known that in CD there are other micronutrient deficiencies (copper, zinc, folic acid and vitamins A, D, E, K, B6 and B12) as a consequence of the malabsorption [28]. Gluten is found in cereals rich in the referred micronutrients, so a GFD may predispose to its deficiency [29]. In our study, children with CD who followed a GFD for more than 6 months had a lower intake of folic acid, magnesium and selenium than the control group. These data are consistent with those reported in other studies [30,31,32] that described a lower intake of folic acid, magnesium and selenium, among other micronutrients. Folate intake in children with CD has been poorly investigated, and there are data that indicate lower folate intake with a GFD [33]. One of the factors that could also explain the differences found in the study in relation to micronutrient intake is the pattern of the diet. However, when we evaluate the frequency of consumption of the different food groups, we only found differences in the intake of meat derivatives that was higher in patients with CD who had followed the GFD for at least 6 months, compared with the control group (*p* < 0.020); these results that have also been described previously [26].

Daily energy intake does not meet Recommended Daily Intake for Spanish population [24] since these are around 2000 kcal for children aged 6 to 9 years and 2400 kcal for children aged 10 to 12 years. With respect to food group consumption, as collected by FFQ, CD > 6 consumed more red meat and subproducts, which has been described in CD by other authors [26]. The daily intake of carbohydrates and the energy intake provided by carbohydrates were lower in the CD < 6 group, although not significantly, and in all groups the percentage of energy supplied by carbohydrates reached the recommendations [24]. Several studies have shown that children have a high risk of consuming excess fats, and that this problem may be increased in patients with CD, because GF bakery products have a higher total fat content and saturated fat [34]. This higher fat intake may predispose to suffer from overweight and obesity, although there are no consistent data to support this fact [35]. In our study no increase in total fat intake was observed.

Previous studies [36] revealed, through a skin model derived from fibroblasts, the importance of nutrient restriction in early stages of life, being one of the earliest determining factors that mark accelerated aging. Fibroblasts have been associated with resistance to multiple forms of cellular stress and with DNA repair mechanisms, increased proteastasis, and resistance to cellular senescence. In CD, it is known that there is an uncontrolled activation of the proinflammatory pathway [37] that maintains the production of free radicals that causes oxidative stress through the increase of reactive oxygen species and consequent damage to cellular DNA [38], and it has been documented that chronic inflammation may be involved in at least a third of cancer cases worldwide [39], so it is essential to ensure a correct balance of micronutrients from childhood.

Another problem that is usually described in children with CD is a low fiber intake due to the lower content of it in the flours used for the manufacture of GF products [40]. In the present study, all children covered the recommended daily intakes due to the consumption of GF vegetables and grains with a fiber content similar to wheat [35]. Data from a study conducted in Spanish children with CD also showed that fiber intake was adequate [41].

In general, patients with CD who participated in the present study were well controlled from a nutritional point of view, and the majority (86%) was aware of the dietary recommendations to be followed (strict GFD) and the performance of physical activity. It has been described that higher levels of adherence to a GFD are positively associated with the perceived adoption of healthy behaviors [42]. In fact, among our patients, it seems that low compliance could be related to a lack of nutritional knowledge and that over time they would acquire more information and there would be greater adherence to the GFD. This is achieved through medical visits during the follow-up of the disease and the participation and dedication of the dietitian, essential for the acquisition of knowledge and obtaining positive reinforcement [43,44].

## 5. Strengths and Limitations

The present study sample size was relatively small, and, consequently, the present results must be interpreted with caution. Based on the present findings, it is advisable to recruit a higher number of participants to render the obtained results with more statistical relevance. Moreover, the groups differed in age, weight and sex, but we controlled all the analyses for such covariates. On the other hand, this is the first study exploring the effect of nutritional adequacy of the GFD and the influence of the time following this GFD on iron status.

## 6. Conclusions

In conclusion, CD may be associated with iron deficiency, due to malabsorption, but also as a result of a poor adherence to a balanced GFD; it is not clear what percentage each factor influences. We believe that the participation of dietitians in the management of the disease is necessary to guide the GFD, to make the diet more balanced, as well as to fortify it in iron and other micronutrients such as folate that may be deficient.

## Figures and Tables

**Table 1:** Anthropometric, clinical and sociodemographic characteristics of the study participants.

Variable	Celiac Group (*n* = 68)	Healthy Group (*n* = 43)	*p*
Sex (female, *n* (%))	52 (76.5)	20 (46.5)	0.014
Age (years)	8.5 (4.1)	10.3 (4.5)	0.025
Anthropometry			
Height (cm)	129.2 (23.5)	142.2 (25.2)	0.006
Weight (kg)	30.5 (12.9)	39.6 (16.4)	0.002
Passive smoker (yes, %)	8.9	25.7	0.031
Time in gluten-free diet (%)			
Not started or less than 6 months	32.9	-	
More than 6 months	67.1	-	
Strictly following gluten-free diet (*n*, (%))	55 (85.9)	1 (1.8)	
Any other celiac family member (%)			
No (or unknown)	35.9	-	
Siblings or parents	46.9	-	
Cousins or uncles	6.3	-	
Various degrees of consanguinity	10.9	-	
Meet physical activity recommendations	75.5	88.9	0.161

Values shown as mean (standard deviation) unless otherwise indicated.

**Table 2:** Differences between healthy control children, patients with a gluten-free diet in the last 6 months and patients with a gluten-free diet less than 6 months on serum hematological and biochemical anemia-related markers.

Variable	Less than 6 Months on Gluten-Free Diet (*n* = 21)	More than 6 Months on Gluten-Free Diet (*n* = 47)	Healthy Control Group (*n* = 43)	*p*
Erythrocytes (millions)	4.74 (0.08) ^a^	4.80 (0.05) ^b^	4.99 (0.05) ^ab^	0.016
Hematocrit (%)	38.6 (0.70) ^a^	40.0 (0.44)	41.0 (0.49) ^a^	0.031
Hemoglobin (mg/dL)	13.2 (0.21)	13.4 (0.14)	13.9 (0.15)	0.046
Erythrocyte corpuscular volume (fL)	82.6 (1.28)	83.2 (0.80)	81.8 (0.89)	0.521
Hemoglobin corpuscular volume (fL)	28.3 (0.47)	28.0 (0.29)	27.9 (0.33)	0.744
RDW	13.3 (0.20)	13.7 (0.13)	13.5 (0.14)	0.130
Iron (µg/dL)	75.7 (8.0)	77.3 (4.5)	89.1 (5.4)	0.226
Ferritin (µg/dL)	44.3 (10.1)	41.9 (5.3)	50.3 (6.5)	0.618
Transferrin (mg/dL)	319.6 (13.3)	292.3 (8.4)	285.5 (11.6)	0.161
TSH (UI/L)	2.42 (0.25)	2.71 (0.17)	2.23 (0.18)	0.150
Thyroxine (µg/dL)	0.97 (0.03)	0.93 (0.02)	0.95 (0.02)	0.588

Values shown as mean (standard error); TSH: thyroid-stimulating hormone; RDW: red blood cell distribution width. Model adjusted for sex, age and body weight. ^a,b^ Common superscript in a same row indicates a significant difference (*p* < 0.05) between the groups. Pairwise comparisons were performed with Bonferroni’s adjustment.

**Table 3:** Differences between healthy control children, patients with a gluten-free diet in the last 6 months and patients with a gluten-free diet less than 6 months on dietary intake outcomes.

Variable	Less than 6 Months on Gluten-Free Diet (*n* = 21)	More than 6 Months on Gluten-Free Diet (*n* = 47)	Healthy Control Group (*n* = 43)	*p*
Dietary intake				
Energy intake (Kcal/day)	1790 (150.5)	1814 (75.1)	1870 (88.9)	0.861
Fat (g/day)	60.3 (8.4)	78.1 (4.2)	75.9 (5.0)	0.171
Protein (g/day)	68.8 (7.5)	70.0 (3.5)	77.0 (4.2)	0.409
Fiber (g/day)	18.8 (1.7)	14.2 (0.8)	15.7 (1.0)	0.056
Carbohydrates (g/day)	179.3 (19.3)	200.1 (9.94)	212.6 (11.7)	0.376
Folate (µg)	159.8 (17.6) ^a^	159.0 (9.0) ^b^	191.4 (10.6) ^ab^	0.075
Cobalamin (µg)	4.04 (1.99) ^a^	7.50 (1.00) ^a^	6.50 (1.18)	0.289
Calcium (mg)	741.8 (99.7)	732.1 (49.7)	798.1 (58.9)	0.701
Iron (mg)	7.32 (0.99) ^a^	7.61 (0.49) ^b^	10.1 (0.58) ^ab^	0.006
Iodine (µg)	74.4 (9.4)	75.7 (6.3)	88.8 (6.8)	0.313
Magnesium (mg)	174.8 (18.4)	165.6 (9.23) ^a^	206.9 (10.9) ^a^	0.022
Selenium (µg)	52.3 (7.0)	45.5 (3.5) ^a^	68.9 (4.2) ^a^	<0.001
Zinc (mg)	6.50 (0.70)	5.81 (0.35)	7.11 (0.42)	0.070

Values shown as mean (standard error); s, servings; m, month; wk, week. Model adjusted for sex, age and body weight. Upper case ^a,b^ letters in the same row indicate a significant pairwise difference (*p* < 0.05) between groups with the same letter. Bonferroni’s correction for multiple comparisons was applied to analyze pairwise differences.

**Table 4:** Differences between healthy control children, patients with a gluten-free diet in the last 6 months versus less than 6 months on food frequency outcomes.

Variable	Less than 6 Months on Gluten-Free Diet (*n* = 17)	More than 6 Months on Gluten-Free Diet (*n* = 35)	Healthy Control Group (*n* = 36)	*p*
Food frequency				
Chicken (s/m)	9.6 (3.3)	10.9 (2.2)	15.8 (2.3)	0.221
Beef (s/m)	3.7 (0.71) ^a^	3.2 (0.47) ^a^	3.5 (0.47)	0.802
Pork (s/m)	4.9 (1.40)	7.7 (0.94)	6.5 (0.96)	0.256
Cured ham (s/m)	7.1 (2.09) ^ab^	13.7 (1.40) ^a^	13.0 (1.40) ^b^	0.034
Ham (s/m)	7.1 (2.68) ^a^	13.6 (1.79) ^ab^	7.3 (1.81) ^b^	0.030
Tukey(s/m)	10.4 (3.27)	10.3 (2.19)	8.59 (2.21)	0.843
Meat derivatives (s/m)	5.5 (2.81) ^a^	13.5 (1.88) ^ab^	7.0 (1.90) ^b^	0.020
Viscera (s/m)	0.13 (0.19)	0.34 (0.13)	0.11 (0.13)	0.440
White fish (s/m)	3.8 (0.9) ^a^	5.9 (0.7)	7.3 (0.7) ^a^	0.025
Blue fish (s/m)	3.1 (0.7)	4.0 (0.5)	5.1 (0.5)	0.104
Lentils (s/m)	3.5 (0.87)	4.3 (0.58)	3.7 (0.59)	0.661
Grouped fish and seafood (s/m)	14.1 (2.86) ^a^	22.8 (1.89) ^a^	21.4 (1.88)	0.044
Grouped red meat and subproducts (s/m)	28.5 (5.13) ^a^	52.1 (3.43) ^a^	37.4 (3.47)	< 0.001
Olive oil (s/m)	50.1 (10.9)	61.7 (7.35)	68.7 (7.43)	0.407
Nuts (s/m)	6.46 (6.73)	9.33 (4.24)	18.4 (4.33)	0.232

Values shown as mean (standard error); s, servings; m, month; wk, week. Model adjusted for sex, age and body weight. Upper case ^a,b^ letters in the same row indicate a significant pairwise difference (*p* < 0.05) between groups with the same letter. Bonferroni’s correction for multiple comparisons was applied to analyze pairwise differences.

## References

[1] Choung R.S., Larson S.A., Khaleghi S., Rubio-Tapia A., Ovsyannikova I.G., King K.S., Larson J.J., Lahr B.D., Poland G.A., Camilleri M.J. (2017). Prevalence and Morbidity of Undiagnosed CD Froma Community-Based Study. Gastroenterology.

[2] Rubio-Tapia A., Ludvigsson J.F., Brantner T.L., Murray J.A., Everhart J.E. (2012). The Prevalence of CDin the United States. Am. J. Gastroenterol..

[3] Mahadev S., Laszkowska M., Sundström J., Björkholm M., Lebwohl B., Green P.H.R., Ludvigsson J.F. (2018). Prevalence of CDin Patients with Iron Deficiency Anemia—A Systematic Review With Meta-analysis. Gastroenterology.

[4] Bottaro G., Cataldo F., Rotolo N., Spina M., Corazza G.R. (1999). The clinical pattern of subclinical/silent celiac disease: An analysis on 1026 consecutive cases 1. Am. J. Gastroenterol..

[5] Martin-Masot R., Nestares M.T., Diaz-Castro J., López-Aliaga I., Muñoz Alférez M.J., Moreno-Fernández J., Maldonado J. (2019). Multifactorial Etiology of Anemia in CDand Effect of Gluten-Free Diet: A Comprehensive Review. Nutrients.

[6] Murray J.A., Watson T., Clearman B., Mitros F. (2004). Effect of a GFD on gastrointestinal symptoms in celiac disease. Am. J. Clin. Nutr..

[7] Leffler D.A., Dennis M., Hyett B., Kelly E., Schuppan D., Kelly C.P. (2007). Etiologies and Predictors of Diagnosis in Nonresponsive Celiac Disease. Clin. Gastroenterol. Hepatol..

[8] Penagini F., Dilillo D., Meneghin F., Mameli C., Fabiano V., Zuccotti G.V. (2013). GFD in children: An approach to a nutritionally adequate and balanced diet. Nutrients.

[9] Thompson T. (2000). Folate, Iron, and Dietary Fiber Contents of the Gluten-free Diet. J. Am. Diet. Assoc..

[10] Thompson T. (1999). Thiamin, riboflavin, and niacin contents of the gluten-free diet: Is there cause for concern?. J. Am. Diet. Assoc..

[11] Hallert C., Grant C., Grehn S., Grännö C., Hultén S., Midhagen G., Ström M., Svensson H., Valdimarsson T. (2002). Evidence of poor vitamin status in coeliac patients on a GFDfor 10 years. Aliment. Pharmacol. Ther..

[12] Muñoz M., Villar I., García-Erce J.A. (2009). An update on iron physiology. World J. Gastroenterol..

[13] Leung A.K., Chan K.W. (2001). Iron deficiency anemia. Adv. Pediatr..

[14] Repo M., Lindfors K., Mäki M., Huhtala H., Laurila K., Lähdeaho M.L., Saavalainen P., Kaukinen K., Kurppa K. (2017). Anemia and iron deficiency in children with potential celiac disease. J. Pediatr. Gastroenterol. Nutr..

[15] Harper J.W., Holleran S.F., Ramakrishnan R., Bhagat G., Green P.H.R. (2007). Anemia in CD is multifactorial in etiology. Am. J. Hematol..

[16] Kalayci A.G., Kanber Y., Birinci A., Yildiz L., Albayrak D. (2005). The prevalence of coeliac disease as detected by screening in children with iron deficiency anaemia. Acta Paediatr..

[17] Freeman H.J. (2015). Iron deficiency anemia in celiac disease. World J. Gastroenterol..

[18] Annibale B., Severi C., Chistolini A., Antonelli G., Lahner E., Marcheggiano A., Iannoni C., Monarca B., DelleFave G. (2001). Efficacy of GFDalone on recovery from iron deficiency anemia in adult celiac patients. Am. J. Gastroenterol..

[19] Husby S., Koletzko S., Korponay-Szabo I.R., Mearin M.L., Phillips A., Shamir R., Troncone R., Giersiepen K., Branski D., Catassi C. (2012). European Society for Pediatric Gastroenterology, Hepatology, and Nutrition guidelines for the diagnosis of coeliac disease. J. Pediatr. Gastroenterol. Nutr..

[20] Cole T.J., Bellizzi M.C., Flegal K.M., Dietz W.H. (2000). Establishing a standard definition for child overweight and obesity worldwide: International survey. BMJ.

[21] Kapur G., Patwari A.K., Narayan S., Anand V.K. (2003). Iron Supplementation in Children with Celiac Disease. Indian J. Pediatr..

[22] Mataix J.L., Martinez de Victoria E., Montellano M.A., Lopez M., Aranda P.L. (2000). Valoración del Estado Nutricional de la ComunidadAutónoma de Andalucía.

[23] Ruiz M.D., Artacho R. (2011). Guía Para EstudiosDietéticos: ÁlbumFotográfico de Alimentos.

[24] Moreiras O., Carbajal A., Cabrera L., Cuadrado C. (2016). Ingestasdiariasrecomendadas de energía y nutrientespara la poblaciónespañola. Tablas de Composición de Alimentos.

[25] King J.A., Jeong J., Underwood F.E., Quan J., Panaccione N., Windsor J.W., Coward S., de Bruyn J., Ronksley P.E., Shaheen A.A. (2020). Incidence of Celiac Disease Is Increasing Over Time: A Systematic Review and Meta-analysis. Am. J. Gastroenterol..

[26] Lionetti E., Antonucci N., Marinelli M., Bartolomei B., Franceschini E., Gatti S., Catassi G.N., Verma A.K., Monachesi C., Catassi C. (2020). Nutritional status, dietary intake, and adherence to the mediterranean diet of children with CD on a gluten-free diet: A case-control prospective study. Nutrients.

[27] Rajalahti T., Repo M., Kivelä L., Huhtala H., Mäki M., Kaukinen K., Kurpaa K. (2017). Anemia in pediatric celiac disease: Association with clinical and histoogical features and response to gluten-free diet. J. Pediatr. Gastroenterol. Nutr..

[28] Di Nardo G., Villa M.P., Conti L., Ranucci G., Pacchiarotti C., Principessa L., Rauci U., Parisei P. (2019). Nutritional deficiencies in children with celiac disease resulting from a gluten-free diet: A systematic review. Nutrients.

[29] Melini V., Melini F. (2019). Gluten-free diet: Gaps and needs for a healthier diet. Nutrients.

[30] Kautto E., Ivarsson A., Norström F., Högberg L., Carlsson A., Hörnell A. (2014). Nutrient intake in adolescent girls and boys diagnosed with coeliac disease at an early age is mostly comparable to their non-coeliac contemporaries. J. Hum. Nutr. Diet..

[31] Balamtekin N., Aksoy Ç., Baysoy G., Uslu N., Demir H., Köksal G., Saltık-Temizel İ.N., Özen H., Gürakan F., Yüce A. (2015). Is compliance with GFDsufficient? Diet composition of celiac patients. Turk. J. Pediatr..

[32] Alzaben A., Turner J., Shirton L., Samuel T.M., Persad R., Mager D. (2015). Assessing Nutritional Quality and Adherence to the GFDin Children and Adolescents with Celiac Disease. Can. J. Diet. Pract. Res..

[33] Babio N., Alcázar M., Castillejo G., Recasens M., Martínez-Cerezo F., Gutiérrez-Pensado V., Masip G., Vaqué C., Vila-Martí A., Torres-Moreno M. (2017). Patients With Celiac Disease Reported Higher Consumption of Added Sugar and Total Fat Than Healthy Individuals. J. Pediatr. Gastroenterol. Nutr..

[34] Miranda J., Lasa A., Bustamante M.A., Churruca I., Simón E. (2014). Nutritional differences between a gluten-free diet and a diet containing equivalent products with gluten. Plant Foods Hum. Nutr..

[35] Sue A., Dehlsen K., Ooi C.Y. (2018). Paediatric Patients with Coeliac Disease on a Gluten-Free Diet: Nutritional Adequacy and Macro- and Micronutrient Imbalances. Curr. Gastroenterol. Rep..

[36] Salmon A.B., Dorigatti J., Huber H.F., Li C., Nathanielsz P.W. (2018). Maternal nutrient restriction in baboon programs later-life cellular growth and respiration of cultured skin fibroblasts: A potential model for the study of aging-programming interactions. GeroScience.

[37] Diaz-Castro J., Muriel-Neyra C., Martin-Masot R., Moreno-Fernandez J., Maldonado J., Nestares T. (2019). Oxidative stress, DNA stability and evoked inflammatory signaling in young celiac patients consuming a gluten-free diet. Eur. J. Nutr..

[38] Nilsen E.M., Lundin K.E., Krajci P., Scott H., Sollid L.M., Brandtzaeg P. (1995). Gluten specific, HLA-DQ restricted T cells from coeliac mucosa produce cytokines with Th1 or Th0 profile dominated by interferon gamma. Gut.

[39] Coussens L.M., Werb Z. (2002). Inflammation and cancer. Nature.

[40] Martin J., Geisel T., Marech C., Krieger K., Stein J. (2013). Inadequate nutrient intake in patient with celiac disease. Results from a German dietary survey. Digestion.

[41] BallesteroFernández C., Varela-Moreiras G., Úbeda N., Alonso-Aperte E. (2019). Nutritional status in spanish children and adolescents with celiac disease on a gluten free diet compared to non-celiac disease controls. Nutrients.

[42] Fueyo-Díaz R., Magallón-Botaya R., Gascón-Santos S., Asensio-Martínez Á., Palacios-Navarro G., Sebastián-Domingo J.J. (2019). The effect of self-efficacy expectations in the adherence to a gluten free diet in celiac disease. Psychol. Health..

[43] Grupo de trabajo del Protocolopara el diagnósticoprecoz de la enfermedadcelíaca (2018). Protocolo Para el DiagnósticoPrecoz de la EnfermedadCelíaca.

[44] Fok C.Y., Holland K.S., Gil-Zaragozano E., Paul S.P. (2016). The role of nurses and dietitians in managing paediatric coeliac disease. Br. J. Nurs..

